# Prognostic nomograms for predicting overall survival and cancer-specific survival in patients with angiosarcoma, a SEER population-based study

**DOI:** 10.1038/s41598-022-07444-5

**Published:** 2022-03-03

**Authors:** Ting Jiang, Zixiang Ye, Tianyu Shao, Yiyang Luo, Binbin Wang

**Affiliations:** 1 Department of Oncology, The First Affiliated Hospital of Zhejiang Chinese Medical University, Hangzhou, 310006 Zhejiang China; 2 Department of Cardiology, Peking University China-Japan Friendship School of Clinical Medicine, Beijing, 100029 China; 3 First Clinical Medical College, Zhejiang Chinese Medical University, Hangzhou, 310053 Zhejiang China

**Keywords:** Cardiology, Diseases, Oncology

## Abstract

Angiosarcoma (AS) is a kind of highly aggressive cancer with high occurrence and mortality rates. This study aimed to establish a comprehensive and validated prognostic nomogram with various clinical indicators in non-metastatic AS patients after surgery. Data of non-metastatic AS patients diagnosed after surgery between 2010 and 2015 was retrieved from the surveillance epidemiology and end results database. Univariate and multivariate Cox proportional hazards regression analysis were performed to identify the independent prognostic factors associated with survival to construct the predictive nomogram of 3- and 5-year overall survival (OS) and cancer-specific survival (CSS) rates. Concordance-index (C-index), calibration plots and receiver operating characteristic (ROC) curves were applied to evaluate the predictive ability of the nomograms. 251 patients in total were divided into the training group (N = 177) and the validation group (N = 74). After the multivariate Cox regression analysis, gender, AJCC stage group 7th ed, T, N stage 7th ed, histologic grade and primary site were statistically identified as independent factors with OS and CSS (*P* < 0.05). We incorporated the significant factors above and age into nomograms. The C-index of the nomograms for OS and CCS in the training cohort was 0.757 (95%CI 0.697–0.817) and 0.762 (95%CI 0.702–0.822), meanwhile, the C-index of those in the validation cohort was 0.749 (95%CI 0.668–0.830) and 0.756 (95%CI 0.676–0.836) respectively. The results of calibration plots and ROC curve showed the nomograms qualified to measure the risk and prognosis. Our study has developed novel and practical nomograms for predicting prognosis in patients with non-metastatic AS after surgery contributing to cancer management.

## Introduction

Soft tissue sarcoma is a highly aggressive human malignancy, among which the rare Angiosarcoma (AS) accounted for less than 1%^1,2^. AS, originating from blood or lymphatic vessels, can occur in any position of the body with high proportions of recurrence and distant metastasis after surgery. It has been reported that the median overall survival (OS) varied from 30 to 50 months for AS patients, resulting in a poor prognosis^3^. Previous study confirmed the absence of surgical excision was correlated with the poor prognosis of AS patients thus surgery is the preferred therapy for AS patients with no metastasis^4^, however, they would face the relapse and distant metastasis. It has been demonstrated that AS patients with elder age^5^, prior radiation therapy^6^, tumor size > 5 cm, primary site from Liver^7^ related to a worse prognosis. However, these factors can only serve as a single predictive indicator, which led to limited effect in comprehensively assessing the prognosis of AS. Therefore, a more integrated system combining multi-factors is needed.

Nomogram is widely applied as a predictive tool that incorporates multiple hazardous variables to forecast the disease prognosis in a visualized manner^8^. However, limited data on this issue in non-metastatic AS patients after surgery has been published. Under the circumstances of various clinical characteristics in each patient with AS, an applicable, accurate and fully-varied prognostic nomogram is imperatively warranted. Hence, by utilizing the data from surveillance epidemiology and end results (SEER) database, we present this study to predict the independent factors in non-metastatic AS after surgery and to construct a prognostic model for better guiding the clinical treatment and follow-up schedules for patients.

## Materials and methods

### Patients enrolled from SEER

The basic information of post-operative AS patients with no metastasis including clinical characteristics and survival data was extracted from the database which is supported by the National Cancer Institute named SEER using the SEER Stat software (version 8.3.6; National Cancer Institute, USA). In line with the International Classification of Disease for Oncology Third Edition (ICD-O-3: 9120/3), the eligible AS patients were screen out. The diagnosis year of patients was during 2010 and 2015. The inclusion criteria of this study were: (1) AS was the unique primary cancer (2) diagnostic confirmation via positive histology; (3) active follow-up; (4) with hospital inpatient/outpatient or clinic reporting source; (5) available data on gender, age, race, survival time, American Joint Committee on Cancer (AJCC) TNM stage, histologic grade, primary cancer site and other details; (6) surgery had been performed with no metastasis. Patients not appropriate for the inclusion criteria were excluded in this study.

All of the patients enrolled were randomly assigned to the training cohort and validation cohort in the ratio of 7:3. All the data in SEER can be downloaded freely with a publicly available ethics approval. All methods were carried out in accordance with relevant guidelines and regulations.

### Clinical variables

Data of demographic and clinical variables such as age, race, gender, primary site, histologic grade, TNM stage, survival data, cause of death, and survival status was acquired from the SEER program. Overall survival (OS) was the primary interest in this study calculated from the diagnosis to the final follow-up or all-cause death. Cancer-specific survival (CSS), the second endpoint defined as the period from the diagnosis to the death due to cancer progression. By using X-tile bioinformatics software (Yale University, USA, Version 3.6.1), the optimal cut-off value of age was 50- and 83-years.

### Statistical analysis

The baseline of categorical variables was compared by the Chi-square test and the Kaplan–Meier method was utilized for survival analysis. Univariate and multivariable Cox regression analysis were presented to identify factors significantly related to survival. Nomograms to predict the 3- and 5-OS and CSS rates were constructed by integrating those independent risk factors from multivariable Cox results. The nomograms were validated both internally (based on the training group) and externally (based on the validation group). The C-index (Harrell’s concordance index) was applied to estimate the predictive ability of nomograms. In general, if a C‐index value is larger than 0.7, the predictive model is regarded as a good prediction^9^. In addition, a receiver operating characteristic (ROC) curve was built to calculate the corresponding areas under the curve (AUC) value. It was universally acknowledged that the larger the AUC value was, the more significance the parameter accounted for. The calibration plot was undertaken to compare the nomogram‐predictive survival probabilities and the actual one with a 45° reference line. All of data analysis was conducted by R Software (Version 4.0.1, Vienna, Austria) with relevant packages such as rma, survival, stdca, cmprsk. Statistical significance in Univariate analysis was set with *P* < 0.2 while *P* < 0.05 was limited for multivariate analysis.

## Results

### Input data from SEER

**Figure 1:**
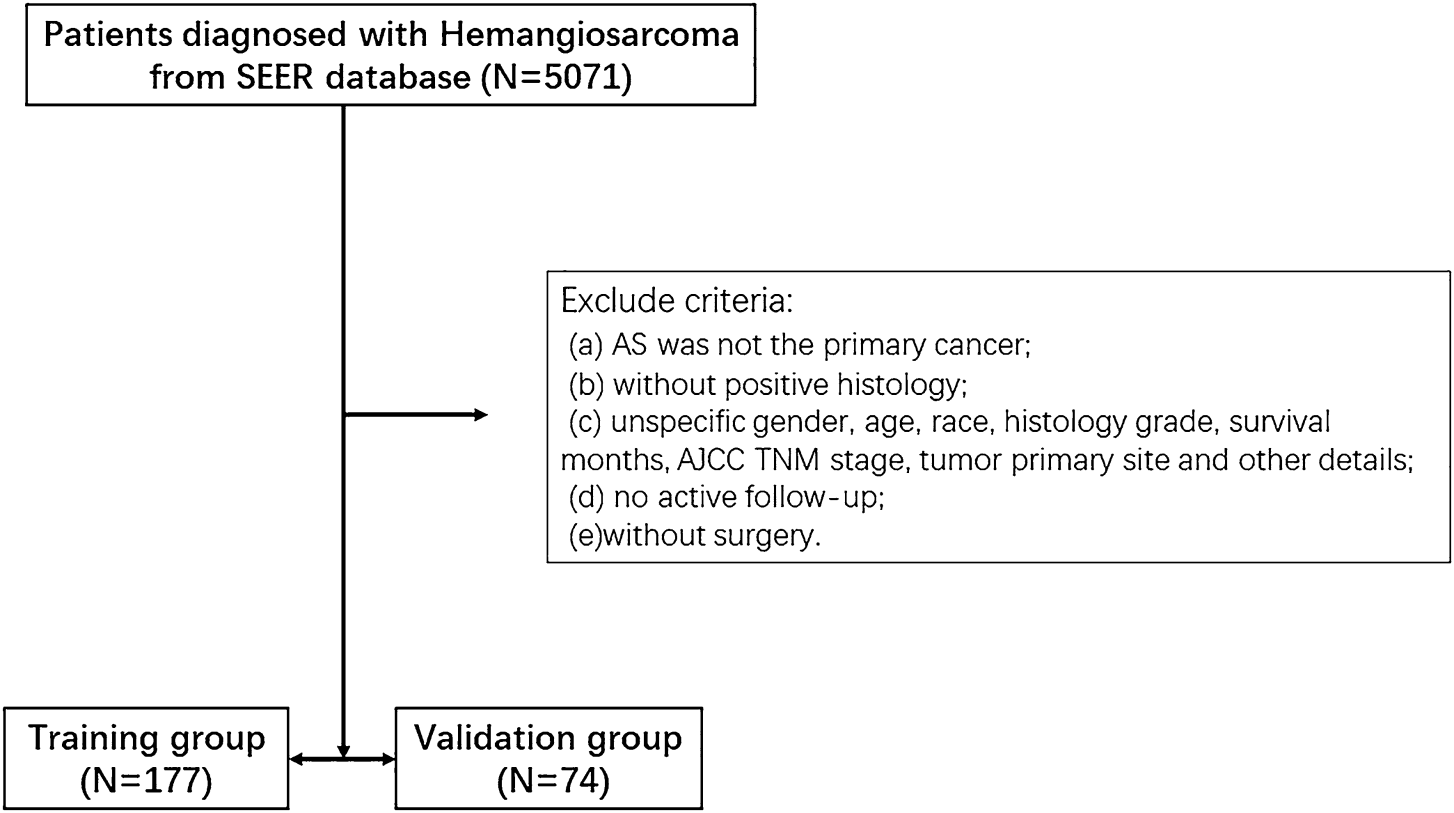
Patients’ selection procession flow chart.

Based on the inclusion and exclusion criteria, 251 of the 5071 patients registered in the SEER database between 2010 and 2015 were enrolled. The patients’ exclusion criteria were as follows (Fig. 1): (a) AS was not the primary cancer; (b) without positive histology; (c) unspecific gender, age, race, histology grade, survival months, AJCC TNM stage, tumor primary site and other details; (d) no active follow-up; (e)without surgery. Finally, 251 patients were brought into this study randomly divided into the training group (N = 177) and the validation group (N = 74). The demographic and clinicopathologic information of the patients in training group was displayed in Table 1. The number of patients aged < 50, 50–82, ≥ 83 years were 33 (13.1%), 181 (72.2%), 37 (14.7%). The majority population were White people (219, 87.3%) and the rest were Black people (17, 6.8%) with only a minority of other races (15, 5.9%). The number of people with primary site soft tissue, heart mediastinum, peritoneum and bone were 219 (87.3%), 17 (6.7%), 5 (2%) and 10 (4%), respectively. The numbers of people with histologic grades I (well-differentiated), II (Moderately differentiated), III (Poorly differentiated), and IV (Undifferentiated) were 25 (10%), 34 (13.5%), 98 (38.7%), and 94 (37.8). The ratio of people with clinical stages IA, IB, IIA, IIB, and III were 30 (12%), 23 (9.2%) and 79 (31.5%), 15 (6%), 104 (41.3%), respectively. (Table 1).

**Table 1:** Basic characteristic of AS patients from SEER.

Variables	Total (n = 251)	Training (n = 177)	Validation(n = 74)	*P*
**Age, n (%)**				0.24
< 50	33 (13.1)	25 (14.1)	8 (10.8)	
50–82	181 (72.2)	129 (72.9)	52 (70.3)	
≥ 83	37 (14.7)	23 (13)	14 (18.9)	
**Race, n (%)**				1
White	219 (87.3)	154 (87)	65 (87.8)	
Black	17 (6.8)	12 (6.8)	5 (6.8)	
Other	15 (5.9)	11 (6.2)	4 (5.4)	
**Gender, n (%)**				0.78
Male	117 (46.6)	81 (45.8)	36 (48.6)	
Female	134 (53.4)	96 (54.2)	38 (51.4)	
**Primary Site, n (%)**				0.63
SoftTissue	219 (87.3)	152 (85.9)	67 (90.5)	
HeartMediastinum	17 (6.7)	14 (7.9)	3 (4.1)	
Peritoneum	5 (2)	3 (1.7)	2 (2.7)	
Bone	10 (4)	8 (4.5)	2 (2.7)	
**Grade, n (%)**				0.18
Grade I	25 (10)	14 (7.9)	11 (14.9)	
Grade II	34 (13.5)	27 (15.3)	7 (9.5)	
Grade III	98 (38.7)	73 (40.7)	25 (33.8)	
Grade IV	94 (37.8)	63 (36.1)	31 (41.8)	
**Stage, n (%)**				0.82
IA	30 (12)	20 (11.3)	10 (13.5)	
IB	23 (9.2)	15 (8.5)	8 (10.8)	
IIA	79 (31.5)	58 (32.8)	21 (28.4)	
IIB	15 (6)	12 (6.7)	3 (4.1)	
III	104 (41.3)	72 (40.7)	32 (43.2)	
**AJCC T stage (7th), n (%)**				0.93
T1a	49 (19.5)	33 (18.6)	16 (21.6)	
T1b	74 (29.5)	54 (30.5)	20 (27)	
T2a	41 (16.3)	27 (15.3)	14 (18.9)	
T2b	72 (28.7)	53 (29.4)	19 (25.7)	
T3	1 (0.4)	1 (0.6)	0 (0)	
TX	14 (5.6)	9 (5.6)	5 (6.8)	
**AJCC N stage (7th), n (%)**				0.86
N0	226 (90)	159 (89.8)	67 (90.5)	
N1	25 (10)	18 (10.2)	7 (9.5)	

*AS* angiosarcoma, *SEER* surveillance epidemiology and end results.

### Construction of nomogram

**Figure 2:**
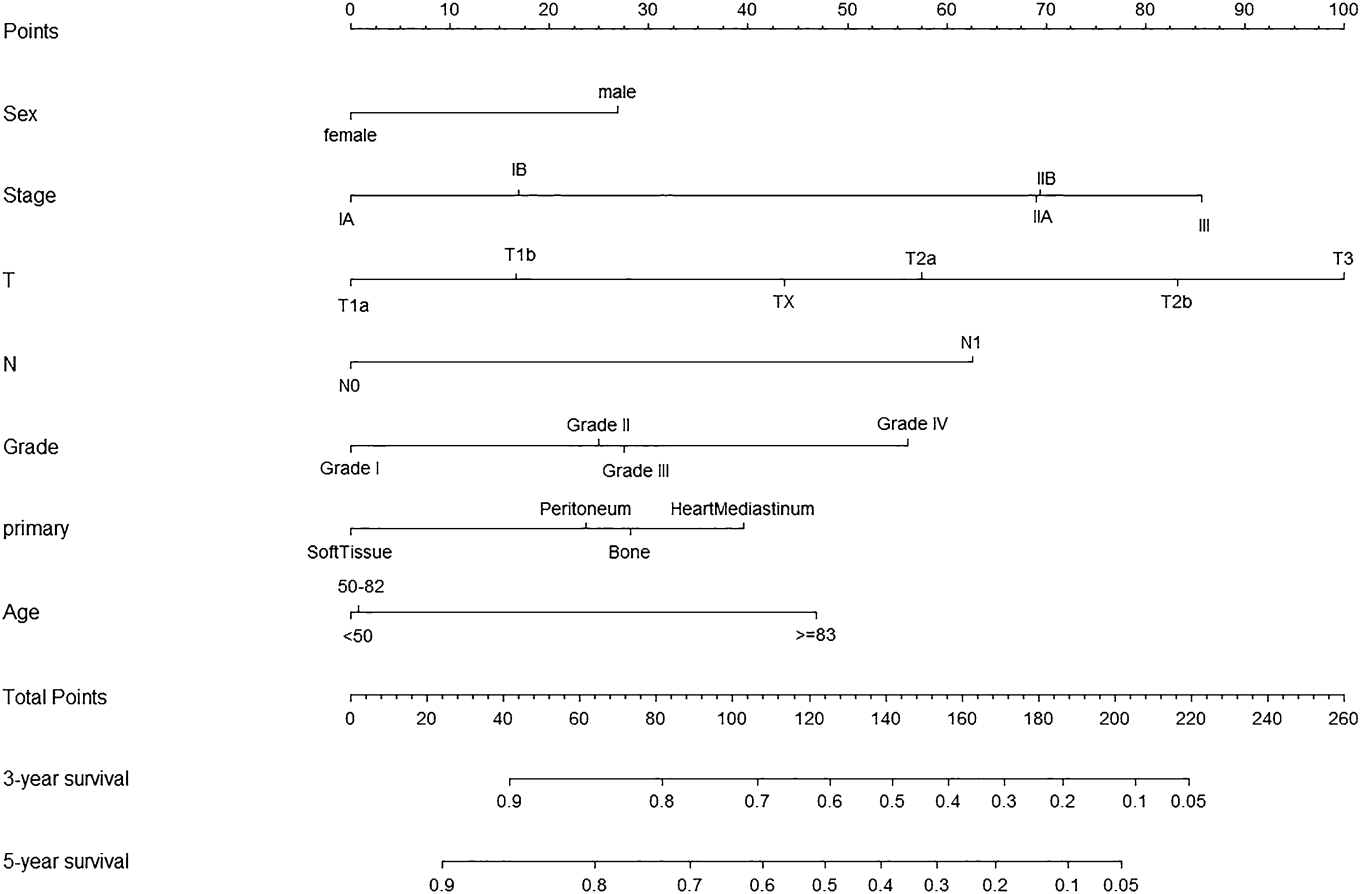
Nomograms to predict 3- and 5-year OS of angiosarcoma. *OS* overall survival.

**Figure 3:**
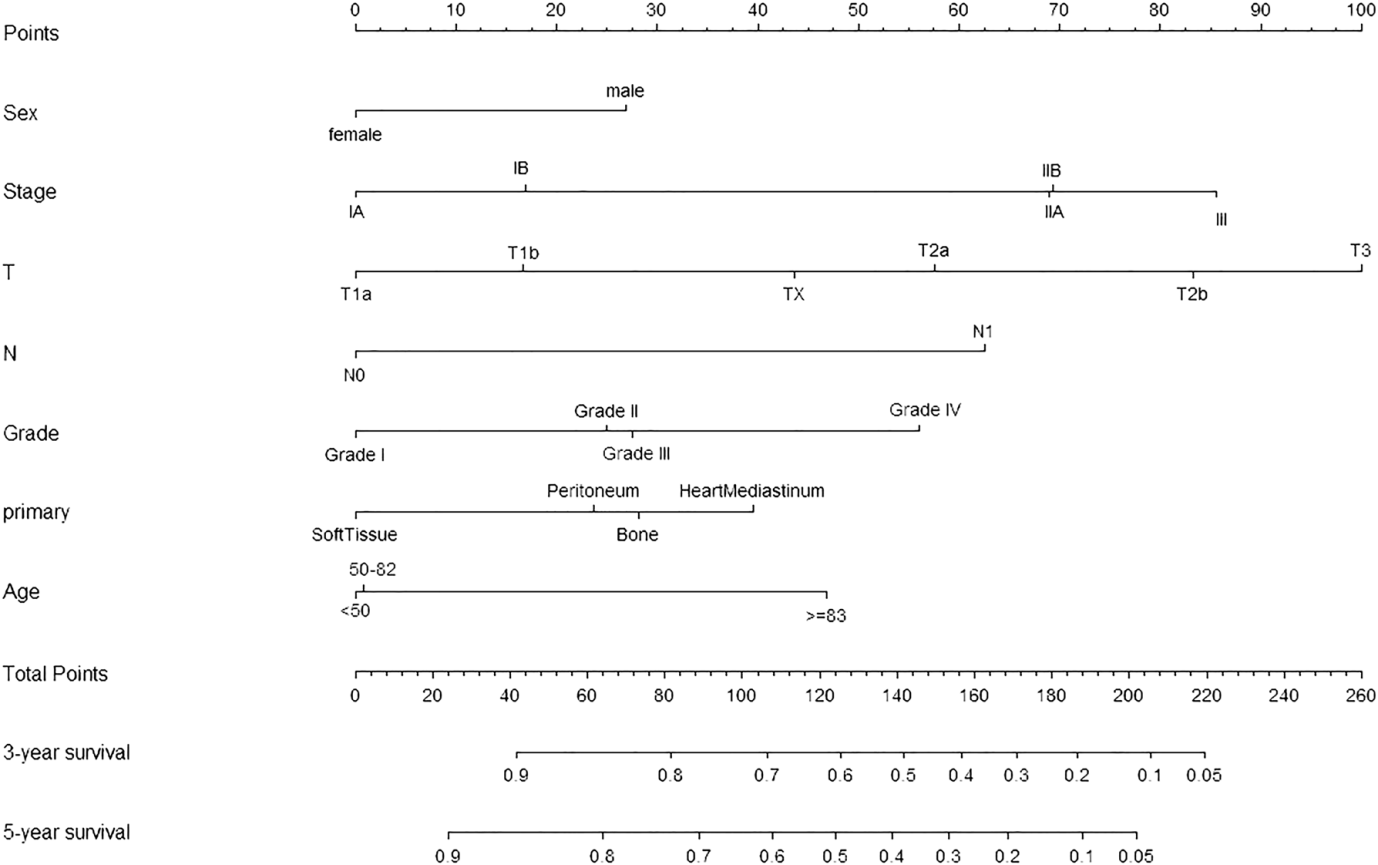
Nomograms to predict 3- and 5-year CSS of angiosarcoma. *CSS* cancer-specific survival.

According to the COX analysis and threshold value, the variables, including gender, histologic grade, AJCC stage group 7th ed, T and M stage 7th ed and primary site were statistically identified with OS in univariate COX regression as well as in the multivariate COX analysis (Table 2). Age was found no significant in univariate COX but was significant in multivariate COX analysis. Nonetheless, age was an acknowledged influencing factor in diverse kinds of diseases and lots of studies have arrived at the agreement that age was significantly associated with survival in AS patients so we included age in the nomogram^5,10–12^. Aiming to predict the 3- and 5-year OS rates, the nomograms were created with these independent factors (Fig. 2). Additionally, the independent variables mentioned above also contributed significantly to the CSS (Table 3), which were utilized to draw the nomogram for CSS prediction (Fig. 3).

**Table 2:** Analysis for OS based on training group by univariable and multivariable Cox proportion regression.

Characteristics	Univariate analysis		Multivariate analysis	
	HR (95%CI)	*P* value	HR (95%CI)	*P* value
**Gender**				
Male	Reference		Reference	
Female	0.58 (0.39–0.87)	0.009	0.60 (0.39–0.96)	0.033
**Age**				
< 50	Reference		Reference	
50–82	1.23 (0.41–1.31)	0.297	1.59 (0.44–2.03)	0.968
≥ 83	1.57 ( 0.76–3.24)	0.216	2.38 (0.97–5.85)	0.048
**Race**				
White	Reference		–	
Black	0.85 (0.43–0.68)	0.659	–	–
Other	0.64 (0.28–0.47)	0.294	–	–
**Grade**				
Grade I; Well differentiated	Reference		Reference	
Grade II; Moderately differentiated	3.85 (1.12–13.18)	0.031	1.59 (0.39–6.48)	0.510
Grade III; Poorly differentiated	3.20 (0.98–10.47)	0.053	1.67 (0.38–7.38)	0.492
Grade IV; Undifferentiated	4.36 (1.34–14.12)	0.013	2.87 (0.70–11.64)	0.039
**AJCC stage group (7th)**				
IA	Reference		Reference	
IB	1.26 (0.24–2.42)	0.651	1.27 (0.04–1.56)	0.145
IIA	1.81 (0.80–4.09)	0.153	1.36 (0.52–3.55)	0.520
IIB	3.67 (1.39–9.67)	0.008	2.00 (0.19–5.31)	0.993
III	2.07 (0.93–4.63)	0.073	2.37 (0.07–1.86)	0.231
**AJCC T stage (7th)**				
T1a	Reference		Reference	
T1b	1.13 (0.51–1.69)	0.822	1.23 (0.67–1.80)	0.348
T2a	0.85 (0.40–1.76)	0.663	2.15 (0.52–8.83)	0.284
T2b	1.73 (0.97–3.06)	0.058	3.50 (0.99–12.32)	0.045
T3	1.68 (0.22–12.69)	0.610	4.80 (0.26–6.97)	0.288
TX	0.39 (0.11–1.35)	0.141	1.66 (0.29–9.23)	0.561
**AJCC N stage (7th)**				
N0	Reference		Reference	
N1	1.54 (0.85–2.77)	0.146	3.24 (1.29–8.16)	0.012
**Primary site**				
Soft tissue	Reference		Reference	
Heart Mediastinum	2.27 (1.17–4.40)	0.015	2.10 (0.81–5.45)	0.026
Peritoneum	3.35 (1.05–10.66)	0.040	1.56 (0.37–6.53)	0.540
Bone	1.30 (0.52–3.23)	0.567	1.69 (0.54–5.30)	0.361

*OS* overall survival.

**Table 3:** Analysis for CSS based on training group by Univariable and multivariable Cox proportion regression.

Characteristics	Univariate analysis		Multivariate analysis	
	HR (95%CI)	*P* value	HR (95%CI)	*P* value
**Gender**				
Male	Reference		Reference	
Female	0.56 (0.39–0.87)	0.037	0.68 (0.42–1.09)	0.010
**Age**				
< 50	Reference		Reference	
50–82	1.23 (0.41–1.31)	0.014	1.08 (0.53–1.81)	0.027
≥ 83	1.57 ( 0.76–3.24)	0.206	1.53 (0.59–3.92)	0.704
**Race**				
White	Reference		–	
Black	0.75 (0.23–2.41)	0.632	–	–
Other	0.52 (0.12–2.14)	0.366	–	–
**Grade**				
Grade I; Well differentiated	Reference		Reference	
Grade II; Moderately differentiated	4.10 (1.12–13.18)	0.191	3.29 (0.34–31.52)	0.307
Grade III; Poorly differentiated	5.61 (1.34–14.12)	0.092	4.32 (0.49–38.04)	0.348
Grade IV; Undifferentiated	8.50 (0.98–10.47)	0.035	8.22 (0.97–69.01)	0.007
**AJCC stage group (7th)**				
IA	Reference		Reference	
IB	1.26 (0.24–2.42)	0.651	1.07 (0.12–4.69)	0.894
IIA	1.81 (0.80–4.09)	0.153	1.31 (0.35–4.81)	0.020
IIB	2.07 (0.93–4.63)	0.073	1.71 (0.46–6.28)	0.031
III	3.67 (1.39–9.67)	0.008	4.64 (1.07–19.96)	0.133
**AJCC T stage (7th)**				
T1a	Reference		Reference	
T1b	1.13 (0.51–1.69)	0.595	1.14 (0.40–1.35)	0.643
T2a	1.58 (0.40–1.76)	0.718	1.46 (0.59–0.63)	0.522
T2b	1.68 (0.22–12.69)	0.996	1.68 (0.16–0.04)	0.996
T3	1.73 (0.97–3.06)	0.020	2.53 (1.05–0.09)	0.438
TX	0.39 (0.11–1.35)	0.536	1.31 (0.91–0.87)	0.682
**AJCC N stage (7th)**				
N0	Reference		Reference	
N1	1.89 (0.92–3.88)	0.079	1.60 (0.90–2.85)	0.016
**Primary site**				
Soft tissue	Reference		Reference	
Heart mediastinum	3.58 (1.73–7.41)	< 0.001	2.53 (1.26–5.04)	0.232
Peritoneum	2.01 (0.27–14.73)	0.488	1.51 (0.22–4.21)	0.918
Bone	1.50 (0.46–4.89)	0.494	1.78 (0.47–6.75)	0.069

*CSS* cancer-specific survival.

### Nomogram validation

**Figure 4:**
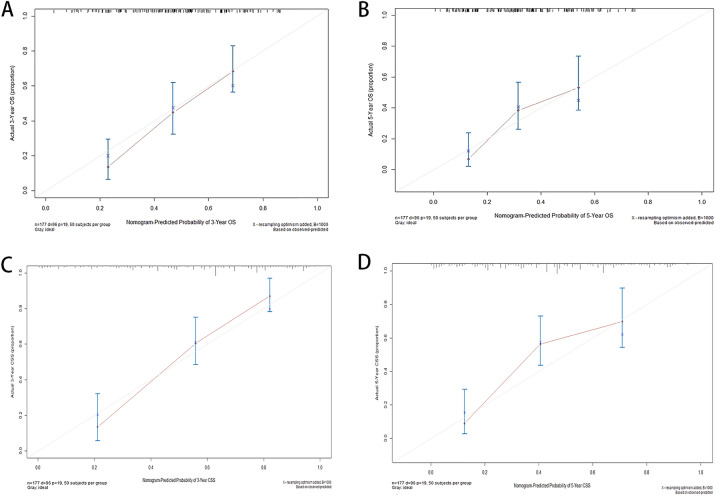
The calibration curve to predict 3-(**A**) and 5-year (**B**) OS and 3-(**C**) and 5-year (**D**) CSS of training sets. *OS* overall survival, *CSS* cancer-specific survival.

**Figure 5:**
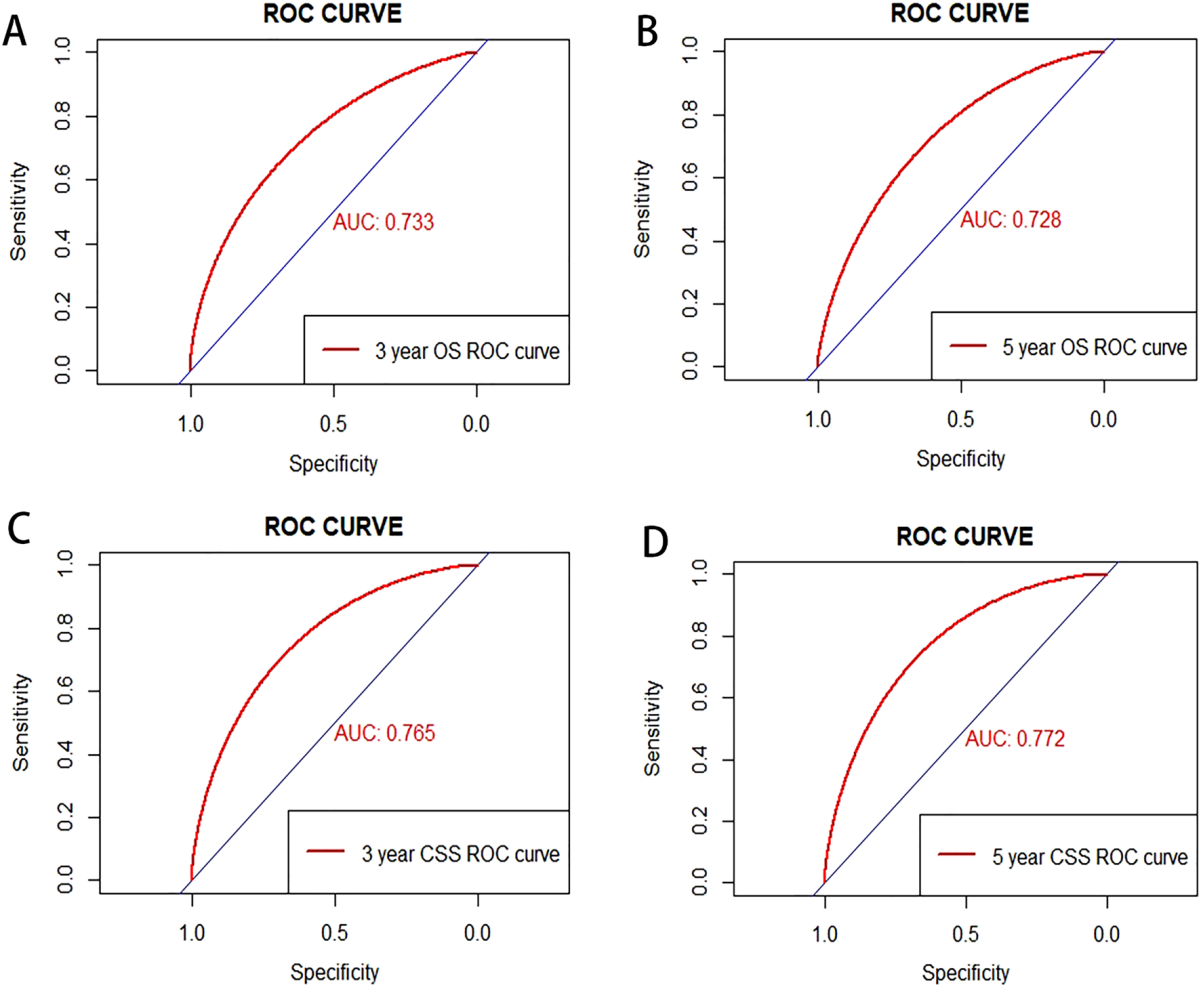
Receiver operating characteristic (ROC) analyses of the nomogram to predict 3-(**A**) and 5-year (**B**) OS in training sets and 3-(**C**) and 5-year (**D**) CSS in training sets. *ROC* receiver operating characteristic, *OS* overall survival, *CSS* cancer-specific survival.

Internal and external methods were used to validate the performance of our nomograms. The predictive accuracy of the nomograms was assessed by exhibiting C-index. The C-indexes were 0.757 (95%CI 0.697–0.817) and 0.762 (95%CI 0.702–0.822) for OS and CSS subjecting to the internal validation. In the external validation, the C-index for the OS nomogram was 0.749 (95%CI 0.668–0.830), while for the CSS nomogram 0.756 (95%CI 0.676–0.836). The calibration curves for the likelihood of 3 and 5-year OS were demonstrated no obviously deviations from the reference line showing a relatively fair agreement between the model prediction and the observed situations (Fig. 4). The validation results showed that the predicted values of both nomograms were in good agreement. In the ROC curves, the AUC values were also applied to evaluate the predictive performance in training (Fig. 5).

## Discussion

The TNM classification system has been universally used to the clinical prediction of prognosis for patients with Soft tissue sarcomas. Nonetheless, it focused mainly on tumor size, lymph nodes and distant metastasis. Some particular clinical factors closely correlated with the prognosis of AS. Besides, Soft tissue sarcoma included a diverse of histological subtypes among them each is heterogeneous from one another. For these reasons, a more highly-specific and sensitive predictive system for AS is necessary.

As a medical system, the nomogram can both predict the independent risk factors associated with diseases among individuals and assist clinicians to discern those patients with high-risk and then adopt optimal therapeutic regimens for them. X-tile software was used to optimize the cut-off value of the variable, age. It selected 50 and 83 as the cut-off point for age which can help better figure out its potential effects on survival rate. The predicting system ability can be greatly enhanced by adding this factor.

In this study, 251 eligible AS patients from the SEER database were all diagnosed in 2010–2015. People with White race, aged 50–82, diagnosed at stage III, and primary site from soft tissues made up the majority of the investigated population. Based on the univariate and multivariate regression, gender, age, AJCC stage group 7th ed, T and N stage 7th ed, histologic grade and primary site were recognized as the independent predictors of prognosis which were utilized to establish nomograms. The C-index of the model in the internal and external validation were all greater than 0.7 for OS (0.757, 0.749) and CSS (0.762, 0.756). The following ROC curves showed it was qualified to predict the 3-year and 5-year survival rates.

Our study has found a significant relationship between gender and the prognosis of non-metastatic AS patients. Biing Luen Lee reported male sex was the possible predictor of poor prognosis in post-operative AS which was consistent with us. However, some research suggested gender appeared no obvious correlation with OS probably due to the limited sample size^4,13^. As displayed in the nomogram, age was also observed as an independent factor. Retrospective studies by Therese Dettenborn and Clothilde Lindet have reached an agreement on this issue that the elder age (> 70) influenced the AS prognosis adversely^5,10^. The nomograms uncovered the stage T and N were also the risk variables. Richard J. Cassidy et.al illustrated that tumor size ≥ 5 cm functioned as a negative role in Scalp angiosarcomas. Of note, they also found age ≥ 65 related to worse survival^11^. Inna Schott found the negative lymph node status corresponded to the better OS^14^. As for the histologic grade, a recent study concluded AS with grade III histology tended to experience a shorter survival which was consistent with our result^15^. The median survival time of AS was various in different primary sites^16–18^, but which site with the highest mortality has not been well documented. Our study elaborated AS from heart mediastinum harbored the highest risk compared with soft tissue, bone and peritoneum. These findings indicated AS patients with risk factors stated above had better take relevant examinations more frequently.

The raw data was acquired from the SEER database containing abundant demographic characteristics, tumor properties, and large population of survival data from different races and countries which is of good representative population and can better reflect population experience than those studies only from a single center^19^. The results of the nomograms arrived at favorable consistency between predictive survival probabilities and the actual conditions, indicating that this model enabled us to distinguish hazard indicators and predict prognosis precisely.

This nomogram provides important clinical use in AS which are listed as follows. Firstly, Clinicians can more accurately and faster to estimate the survival chances and recognize the personalized risk of AS patients so as to adopt corresponding interventions. For post-operative patients, Individual treatment strategies and the follow-up plans can be optimized based on the different prognosis of patients. For example, a 45-year-old female patient was diagnosed with AS originated from soft tissues. Her tumor size was found T1b with N1 and M0 metastasis. The postoperative pathology was confirmed as grade III with stage IIA. This patient got 175 scores totally in OS nomogram and the corresponding 3- and 5-year predicted OS were 26% and 16%. Following the same method, the predicted CSS can be easily obtained. In this way those at high-risk had better follow up more frequently and detailly. The heterogeneity among patients should be considered when clinicians formulate clinical decision-making, thus, early preventive interventions may be executed to prolong the survival for those high-risk population. Secondly, the soft tissue sarcomas, a variety of huge admixture, contains more than 50 subtypes which are diverse from each other. Our study has specifically targeted AS as a separate category to help its better management. Moreover, some patients with AS cannot be clearly staged for some inevitable reasons which are often staged as Tx. It is usually more difficult for clinicians to predict their prognosis and often required multidisciplinary teamwork. Our study has focused on this special vague cohort to guide better predict prognosis and treatment. In addition, our nomograms have incorporated AJCC stage T, N, and showed great consistency with the training group, supporting this credible model into clinical practice. In short, this nomogram can be utilized as a useful stratification tool for further clinical AS researches.

Still, there were some important clinical variables not available in the SEER database such as chronic lymphedema history, smoking or alcohol drinking habits. Research has reported people having smoking habit or suffering from chronic lymphedema were more susceptible to AS^12,20^. Additionally, specific genes mutation acted oncogenic roles in AS^21,22^ while the SEER database did not contain genetic testing information so we are unable to evaluate these effects. Moreover, the data in this study was derived from the USA population, whether it is applicable to Asians or Europeans needs further evidenced-based study.

## Conclusion

Taken together, our study constructed a novel predictive model to identify the risk in post-operative AS patients with no metastasis. The nomograms were capable to predict 3- and 5-year OS and CSS. These findings can help clinicians in decision-making on personalized treatment strategy.
